# Is ProACS Score Just another Risk Stratification Score, or is it Ready for Implementation in Clinical Practice?

**DOI:** 10.5935/abc.20190122

**Published:** 2019-07

**Authors:** Ana Teresa Timóteo

**Affiliations:** 1 Departamento de Cardiologia - Hospital Santa Marta, Centro Hospitalar Universitário Lisboa Central, Lisboa - Portugal; 2 NOVA Medical School, Lisboa - Portugal

**Keywords:** Cardiovascular Diseases, Acute Coronary Syndrome, Propensity Score, Probability, Risk Factors, Prognosis

Since the first publication of Framingham Risk Score in late 1950’s, derived from data obtained in an observational study to investigate cardiovascular risk factors, several other risk scores were created around the globe and they have been increasingly popular particularly in the last 10-20 years in cardiovascular medicine. The objective of a risk score is to individualize the risk for an individual patient with the use of functional equations based on multiple risk factors. Patients at high risk should be submitted to specific interventions to improve outcome.

Despite this huge increase in risk scoring development, most have not been implemented in clinical practice. In fact, many models showed low predictive performance (as assessed by their calibration and discrimination) and some are poorly reported or were developed using inappropriate methods. For that reason, international guidelines were developed for adequate development and reporting of prediction models.^1^ For that objective to be fulfilled, it should include an adequate selection of the development cohort, adequate methodology for selection of variables and an adequate validation, both internal and external. In fact, external validation is particularly important because it tests the performance of a prediction model in a different independent patient cohort, where they usually perform less well. It can also allow comparison with other risk stratification tools. This step is essential for safe implementation in clinical practice.

Risk stratification is important because populations are heterogeneous and present a large risk spectrum. Presently, there are very expensive and potent treatments, also with increased risk of adverse events, and thus, management decisions must be supported with risk stratification tools, although clinical judgment remains essential in these decisions. Unfortunately, in clinical practice, there is a misperception by the clinician that the use of individual risk indicators is sufficient to predict outcome and there is also a belief that the use of prediction rules may lead to overtreatment and that they are time consuming.^2^ This leads to the treatment-risk paradox that in many cases will result in inadequate treatment and poor outcome in patients at very high risk.

In this journal, Gil et al.^3^ present a paper where they compared several well-known risk stratification scores derived from both randomized clinical trial and registries.^3^ They also included some scores that were developed for other purposes of risk stratification in cardiovascular medicine. The scores that better predicted in-hospital mortality were GRACE, ACTION-Registry-GWTG and ProACS, but for long-term outcome, none of the studied scores was appropriate. This is a retrospective analysis performed in 1,452 patients admitted with an acute coronary syndrome (ACS), 45.1% with ST-elevation myocardial infarction, with in-hospital mortality of 6.5% and 9.9% of death / non-fatal ACS in a one-year follow-up.

ProACS risk score was developed with a very specific purpose of providing a very easy and simple risk score, with immediate variables, that could be applied early, in the first medical contact, to better decide on patient management strategy.^4^ ProACS was developed from one of the largest registries worldwide, the Portuguese Registry of Acute Coronary Syndromes, with more than 45,000 patients included.^5^ In the development study, this score showed good discrimination in all the study cohorts (development, internal and external validation), and similar results were also reported in an independent external validation cohort.^4.6^ Calibration was adequate in all these studies. Results were, however, slightly worst, compared to GRACE risk score and a recommendation was included that this should be used only as an early risk stratification score, with GRACE risk score to be used later after hospital admission. Surprisingly, in the present study, discrimination of ProACS was even high, with similar c-statistics when compared to the most potent risk stratification scores, but calibration was not adequate, particularly in the non-ST elevation ACS cohort.

One important finding is that ProACS risk score is very similar to C-ACS risk score;^7^ however, this one was developed in Canada with different patient characteristics, and had very low discrimination in the Portuguese population, stressing the importance of regional external validation studies. Also, risk scores developed from randomized clinical trials were less useful compared to risk scores developed from registries, in cohorts more representative of real-world populations. Clinical trials cohort is very selective, limiting extrapolation for general populations, patients are usually younger and healthier, with a lower rate of comorbidities and clinical trials do not reproduce practices and strategies used in most centres. For that reason, the external validity of models derived from clinical trials is usually lower, as we observed in the present study.

The main objective of ProACS risk score is to immediately stratify the risk in patients with ACS to decide on the adequate management and thus its main objective is the short-term outcome. Results in longer-term follow up were not adequate. For long-term outcome, the inclusion of other variables, such as renal function, troponins and others are also important.

What should not be forgotten is that for each risk stratification score, impact studies should also be performed after external validation. In fact, external validity does not mean that beneficial clinical effects will be obtained when we use a risk score and it does not give information regarding the possible adverse events. Impact studies are essential and should be studied separately from development and validation, preferably in randomized clinical trials and they are usually not performed. From the above, researchers should spend more time on validation and assessing the impact of existing models instead of developing more models that will most likely never be used in clinical practice. This should be the next step to definitely support the use of ProACS risk score in clinical practice.

From the results presented in this study, ProACS risk score will probably be soon implemented in clinical practice, due to the easy and simple application in clinical practice and high discrimination presented but impact studies are needed.
